# Supplementary material for: Efficacy of Quantitative Pupillary Light Reflex for Predicting Neurological Outcomes in Patients Treated with Targeted Temperature Management after Cardiac Arrest: A Systematic Review and Meta-Analysis
Source: Medicina (Kaunas). 2022 Jun 15;58(6):804. doi: 10.3390/medicina58060804 (PMC9230846; doi:10.3390/medicina58060804)
Supplement: Supplementary file 1 [file medicina-58-00804-s001.zip › medicina-1757228-supplementary.pdf]

Table S1. Comprehensive list presenting the search strategy

EMBASE

1	(return of spontaneous circulation or ROSC).ti,ab. or exp heart arrest/ or cardiac arrest*.ti,ab. or cardiovascular arrest*.ti,ab. or heart arrest*.ti,ab. or cardiopulmonary arrest*.ti,ab. or asystol*.ti,ab. or pulseless electrical activity.ti,ab. or exp ventricular fibrillation/ or exp advanced cardiac life support/ or (advanced cardiac life support or ACLS).ti,ab. or exp cardiopulmonary resuscitation/ or CPR.mp. or cardiopulmonary resuscitation.ti,ab.	225254
2	Hypothermia*.ti,ab. or therapeutic hypothermia.mp. or TTM.ti,ab.	41852
3	1 and 2	9959
4	pupil*.ti,ab. or pupillometry.ti,ab. or pupillometer.ti,ab. or pupillary light reflex. ti,ab.	38656
5	3 and 4	188

MEDLINE

1	(return of spontaneous circulation or ROSC).ti,ab. or exp heart arrest/ or cardiac arrest*.ti,ab. or cardiovascular arrest*.ti,ab. or heart arrest*.ti,ab. or cardiopulmonary arrest*.ti,ab. or asystol*.ti,ab. or pulseless electrical activity.ti,ab. or exp ventricular fibrillation/ or exp advanced cardiac life support/ or (advanced cardiac life support or ACLS).ti,ab. or exp cardiopulmonary resuscitation/ or CPR.mp. or cardiopulmonary resuscitation.ti,ab.	98482
2	Hypothermia*.ti,ab. or therapeutic hypothermia.mp. or TTM.ti,ab.	35423
3	1 and 2	4656
4	pupil*.ti,ab. or pupillometry.ti,ab. or pupillometer.ti,ab. or pupillary light reflex. ti,ab.	30702
5	3 and 4	74

Cochrane library

1	(return of spontaneous circulation or ROSC or cardiac arrest* or cardiovascular arrest* or heart arrest* or cardiopulmonary arrest* or asystol* or pulseless electrical activity or ventricular	10752

	fibrillation or advanced cardiac life support or ACLS or cardiopulmonary resuscitation or CPR):ti,ab,kw	
2	(paramedic cooling or field hypothermia or hypothermia or targeted temperature management or therapeutic hypothermia or hypothermia therapy or whole body cooling):ti,ab,kw	4009
3	#1 and #2	790
4	(pupil* or pupillometry or pupillometer or pupillary light reflex):ti,ab,kw	3711
5	#3 and #4	10

Search for other resources

#1	cardiac arrest or cardiopulmonary resuscitation or CPR or return of spontaneous resuscitation, ROSC, advanced cardiac like support
#2	targeted temperature management or TTM or hypothermia or hypothermia therapy or hypothermia treatment
#3	Pupillometry or pupillary reactivity or pupillary light reactivity or pupillary light reflex or pupillary response
#5	#1 and #2 and #3

Table S2. Characteristics of excluded from meta-analysis

	Study	Reason for exclusion
1	Abend 2010	Irrelevant data (not pupillometry data)
2	Al Thenayan 2008	Irrelevant data (not pupillometry data)
3	Bisschops 2011	Irrelevant data (not pupillometry data)
4	Bouwes 2010	Irrelevant data (not pupillometry data)
5	Bouwes 2012	Irrelevant data (not pupillometry data)
6	Bouwes 2011	Irrelevant data (not pupillometry data)
7	Dhakal 2013	Irrelevant data (not pupillometry data)
8	Dhakal 2014	Irrelevant data (not pupillometry data)
9	Eid 2011	Irrelevant data (not pupillometry data)
10	Fugate 2010	Irrelevant data (not pupillometry data)
11	Greer 2013	Irrelevant population (cardiac arrest patients without TTM)
12	Hamilton 2018	Case report
13	Hansen, 2010	Irrelevant population (cardiac arrest patients with or without TTM)
14	Hanumanthu, 2019	Irrelevant data (not quantitative pupillometry data)
15	Hawkes, 2019	Systematic review
16	Henriksen, 2018	Irrelevant population (cardiac arrest patients with or without TTM)
17	Horn, 2010	Irrelevant data (not quantitative pupillometry data)
18	Karapetkova, 2016	Systematic review
19	Kim, 2019	Irrelevant data (not pupillometry data)
20	Kongpolprom, 2016	Irrelevant data (not pupillometry data)
21	Kongpolprom, 2018	Irrelevant data (not pupillometry data)
22	Leithner, 2015	Irrelevant data (not pupillometry data)
23	Maekawa, 2012	Irrelevant data (not pupillometry data)
24	Maekawa, 2013	Irrelevant population (cardiac arrest patients without TTM)
25	Maia, 2013	Irrelevant data (not pupillometry data)
26	Matthews, 2015	Irrelevant data (not pupillometry data)
27	Matthews, 2018	Irrelevant data (not pupillometry data)
28	Mila, 2016	Irrelevant data (not pupillometry data)
29	Oddo, 2017	Irrelevant data (not pupillometry data)
30	Okada, 2012	Irrelevant data (not pupillometry data)
31	Reynolds, 2015	Irrelevant population (cardiac arrest patients without TTM)
32	Reynolds, 2015	Irrelevant population (cardiac arrest patients with or without TTM)
33	Reynolds, 2014	Irrelevant population (cardiac arrest patients with or without TTM)
34	Reynolds, 2016	Editorial
35	Riker, 2019	Irrelevant data (not quantitative data of light reflex)
36	Riker, 2018	Irrelevant data (not quantitative data of light reflex)
37	Rittenberger, 2010	Irrelevant population (cardiac arrest patients without TTM)
38	Samaniego, 2011	Irrelevant data (not pupillometry data)
39	Sarapuddin, 2015	Irrelevant data (not pupillometry data)
40	Sawyer, 2018	Irrelevant data (not quantitative data of light reflex)
41	Simonis, 2010	Irrelevant data (not pupillometry data)
42	Storm, 2015	Irrelevant data (not pupillometry data)
43	Suys, 2013	Duplicated data (abstract of included study)
44	Thomke, 2013	Irrelevant data (not pupillometry data)
45	Touma, 2011	Irrelevant population (cardiac arrest patients with or without TTM)
46	Zellner, 2010	Irrelevant data (not pupillometry data)

Abbreviation: TTM = targeted temperature management.

Table S3. Baseline characteristics of individuals in the included studies

Author	Outcome	N	OHCA, %	Age, yr	Male, n (%)	Witnessed CA, n(%)	Initial rhythm, n (%)		CPR related time, min				Severity score
							Shockable	Non-shockable	Time to ROSC	CPR duration	No-flow time	Low flow time	
Suys*	Good	23	100	60 (34-81)	34 (68)	-	-	4(17)	15 (34-81)	-	-	-	-
	Poor	27		63 (31-88)		-	-	16(59)	25 (5-50)	-	-	-	-
Heimburger†	Good	27	85	52 (48-64)	67(82)	27(100)	32 (76.2)	3(11)	-	15.0 (10.0-20.0)	0(0-5)	10(5-27)	SAPS II = 46(40-62)
	Poor	55	76	64 (54-75)		48(87.2)	25 (59.5)	25(45)	-	30.0 (20.5-43.8)	0(0-4)	30(15-54)	SAPS II = 71(60-88)
Oddo†	Good	187	-	60 (49-69)	154 (80)	-	-	36 (19)	19(10-29)	-	-	-	-
	Poor	269	-	65 (54-74)	203 (72)	-	-	160 (57)	28(16-41)	-	-	-	-
Riker†	All	55	80	57(48-68)	36 (65)	38(69)	28(51)	--	23(14-34)	-	-	-	-

* Continuous variables are presented as median (minimum-maximum)

†Continuous variables are presented as mean ± SD or median (interquartile range).

Time to ROSC: time from cardiac arrest to return of spontaneous circulation (min)

Abbreviation: OHCA = out-of-hospital cardiac arrest; CPR = cardiopulmonary resuscitation; TTM = targeted temperature management; SAPS = Simplified Physiology Score II, APACHE II = Acute Physiology and Chronic Health Evaluation II

Table S4. Characteristics of targeted temperature management, supportive care in the included studies.

Authors	Outcome	TTM protocol				Sedation, analgesia, and neuromuscular blocking agents during TTM				Supportive care and agent	
		**Targeted temperature, (°C)**	**Maintenance (hour)**	**Rewarming rate (°C/h)**	**device**	**Propofol**	**Midazolam**	**Fentanyl**	**Neuromuscular blocking agents**	**Norepinephrine**	**ECLS, %**
Suys*	Good	33.0	24	-	Artic sun®	-	0.1 mg/kg/h	1.5 μg/kg/h	rocuronium 0.6 mg/kg bolus, if shivering	5.1 (0–44) mg/day	--
	Poor									6.2 (0–177.7) mg/day	-
Heimburger†	Good	33.0	24	0.65	-	2mg/kg/h	-	0.3 μg/kg/h	cisatracurium 0.18mg/kg/h	-	4(15)
	Poor	32.5									16(29)
Oddo†	Good	33 - 199(44%)<br>36 – 257(56%)	24	-	-	1.7 (1.1–2.4) mg/h	0.08 (0.04–0.15) mg/kg/h	0.49 (0.26–1.4) μg/kg/h	-	4.9 (1–15) mg/kg/min	-
	Poor					1.8 (1.3–3.7) mg/h	0.11 (0.06–0.37) mg/kg/h	0.55 (0.14–1.4) μg/kg/h	-	5.4 (1–15) mg/kg/min	-
Riker†	All	33.0	24	-	Artic sun®	10–30 mcg/kg/min	-	20–50 mcg/h	-	-	-

* Continuous variables are presented as median (minimum-maximum)

†Continuous variables are presented as mean ± SD or median (interquartile range).

Time to ROSC: time from cardiac arrest to return of spontaneous circulation (min)

Abbreviation: OHCA = out-of-hospital cardiac arrest; CPR = cardiopulmonary resuscitation; TTM = targeted temperature management; SAPS = Simplified Physiology Score II, APACHE II = Acute Physiology and Chronic Health Evaluation II; ECLS = extracorporeal life support

Table S5. Funding and conflict of interest of the included studies

Authors	Funding	Conflict of interest	Blinding	
			Participant and personnel	Outcome assessment
Suys*	**Yes, but no commercial funding.**<br><br>This work was supported by grants from the Swiss National Science Foundation (to MO and AOR), the European Critical Care Research Network (to MO), and the Gueules Casse´es Foundation (to PB).	**None**	-	**Blinding was not performed or incomplete**<br><br>(Measure of quantitative PLR was conducted by the ICU research nurse)
Heimburger†	-	**None**	-	**Blinding was not performed or incomplete**<br><br>(Measure of quantitative PLR was conducted by one of the authors of the study)
Oddo†	**Yes.**<br><br>**The device manufacturer (Neuroptics®, Irvine, CA, USA) supplied equipment and disposables for the study,** but it did not provide any financial support for the study and had no role in the study design, data collection, analysis, and interpretation, or writing of the report.	**Yes.**<br><br>**Mauro Oddo has received speaker honoraria from Neuroptics®.** All the remaining authors declare no conflicts of interest related to this study.	-	**Adequate blinding of outcome assessors**<br><br>(The ICU clinicians/nurses who performed pupillometry tests were not involved in patient care and the outcome assessors were blinded to quantitative pupillometry data.)
Riker†	**Yes, but no commercial funding.**<br><br>Funded by the Maine Medical Center Research Institute Summer Grant.	**None**	-	**Blinding is not described**<br><br>it is unclear its absence is judged to affect the outcome measurement.

* Continuous variables are presented as median (minimum-maximum)

†Continuous variables are presented as mean ± SD or median (interquartile range).

Time to ROSC: time from cardiac arrest to return of spontaneous circulation (min)

Abbreviation: OHCA = out-of-hospital cardiac arrest; CPR = cardiopulmonary resuscitation; TTM = targeted temperature management; SAPS = Simplified Physiology Score II, APACHE II = Acute Physiology and Chronic Health Evaluation II; PLR=pupillary light reflex

Table S6. Data abstraction and study quality assessment of included studies

**Suys 2014**		
**Method**	**Type of study: a** single-center prospective observational study<br>**Countries where the study was performed:** Switzerland<br>**Date of trial:** June 2012 - June 2013<br>**Follow-up until:** Neurological outcome: 3 months<br>**Pupillometry measurement time**: hospital day 1 and 2	
**Participants**<br>(Data are presented as median and ranges)	**Inclusion:**<br>Comatose patients resuscitated from out of hospital cardiac arrest and treated with targeted temperature management.<br><br>**Exclusion:**<br>Cardiac arrest patient not treated with targeted temperature management<br><br>**The number of participants analyzed:**<br>Good neurological outcome (GNO): 23<br>Poor neurological outcome (PNO): 27<br><br>**Age (years) -** GNO: 60 (34-81) / PNO: 63 (31-88)<br>**Sex (male, %) –** 34(68) in total study population	
**Interventions**<br>(Data are presented as median and ranges)	**Automated pupillometer:**<br>The NeuroLight Algiscan® (IDMED, Marseille, France)<br><br>**Targeted temperature management**<br>Target temperature, °C: 33.0<br>Maintenance (hour): 24<br>Rewarming rate (°C/h): not reported<br>TTM devices: not reported<br><br>**Sedation, analgesia, and neuromuscular blocking agents during TTM**<br>Sedation (midazolam, 0.1 mg/kg/h),<br>analgesia (fentanyl, 1.5 lg/kg/h)<br>neuromuscular blocking agents (rocuronium 0.6 mg/kg bolus, if shivering)<br><br>**Supportive care and agent**<br>Norepinephrine (mg/day): not reported<br>Extracorporeal life support (%) **-** GNO: 4(15) / PNO: 16 (29)	
**Outcomes**	Neurological outcome at 3 months	
**Note**	None	
**Quality in Prognosis Studies (QUIPS)**		
**Bias**	Author' judgment	Support for judgment
**Study participation**	Unclear risk	Key characteristics are not adequately described and Only inclusion criteria were described
**Study Attrition**	Low risk	There were no follow-up losses.
**Prognostic Factor Measurement**	Low risk	A clear definition is provided
**Outcome Measurement**	Low risk	A clear definition of outcome is provided
**Study Confounding**	Unclear risk	Important potential confounders are not partially accounted for in the analysis

**Statistical Analysis and Reporting**	Low risk	There is sufficient presentation of data to assess the adequacy of the analysis.
**Heimburger 2016**		
**Method**	**Type of study: a** single-center prospective observational study<br>**Countries where the study was performed:** France<br>**Date of trial:** January 2014 - April 2015<br>**Follow-up until:** Neurological outcome: 3 months<br>**Pupillometry measurement time**: hospital day 1 and 2	
**Participants**<br>(Data are presented as median and interquartile ranges)	**Inclusion:**<br>Cardiac arrest patients who were 18 years or older and had ROSC after in-hospital or out-of-hospital cardiac arrest<br><br>**Exclusion:**<br>Patients who had a known neurological disorder before cardiac arrest, imminent death within the first 12 h, a known ocular pathology, or opposition to participate in the study from relatives.<br><br>**The number of participants analyzed:**<br>Good neurological outcome (GNO): 27<br>Poor neurological outcome (PNO): 55<br><br>**Age (years) -** GNO: 52 (48-64) / PNO: 64 (54-75)<br>**Sex (male, %) –** 67(82) in total study population	
**Interventions**<br>(Data are presented as median and interquartile ranges)	**Automated pupillometer:**<br>The NeuroLight Algiscan® (IDMED, Marseille, France)<br>**Targeted temperature management**<br>Target temperature, °C: 33.0(GNO) / 32.5(PNO)<br>Maintenance (hour): 24 in both<br>Rewarming rate (°C/h): 0.65 in both<br>TTM devices: not reported<br>**Sedation, analgesia, and neuromuscular blocking agents during TTM**<br>Sedation (propofol, 2 mg /kg/ h)<br>analgesia (sufentanil,0.3ug /kg/ h)<br>neuromuscular blocking agents (cisatracurium, 0.18 mg/ kg/ h)<br>**Supportive care and agent**<br>Norepinephrine (mg/day) - GNO: 5.1 (0–44) / PNO: 6.2 (0–177.7)<br>Extracorporeal life support (%): not reported	
**Outcomes**	Neurological outcome at 3 months	
**Note**	None	
**Quality in Prognosis Studies (QUIPS)**		
**Bias**	Author' judgment	Support for judgment
**Study participation**	Low risk	The source population or population of interest is adequately described for key characteristics
**Study Attrition**	Low risk	Reasons for loss to follow-up are provided.
**Prognostic Factor Measurement**	Low risk	Appropriate methods of imputation are used for missing 'PF' data.
**Outcome Measurement**	Low risk	The method and set of outcome measurements are the same for all study participants.

**Study Confounding**	Unclear risk	Important confounders, including treatments, are not partially measured
**Statistical Analysis and Reporting**	Low risk	There is sufficient presentation of data to assess the adequacy of the analysis.
**Oddo 2018**		
**Method**	**Type of study: a** multicenter prospective observational study<br>**Countries where the study was performed:** Switzerland<br>**Date of trial:** January 2015 - March 2017<br>**Follow-up until:** Neurological outcome: 3 months<br>**Pupillometry measurement time**: hospital day 1, 2 and 3	
**Participants**<br>(Data are presented as median and interquartile ranges)	**Inclusion:**<br>Patients were adult subjects (> 18 years) with coma (Glasgow Coma Score ≤ 6) following a cardiac arrest.<br><br>**Exclusion:**<br>Not reported<br><br>**The number of participants analyzed:**<br>Good neurological outcome (GNO): 187<br>Poor neurological outcome (PNO): 269<br><br>**Age (years) -** GNO: 60 (49-69) / PNO: 65 (54-74)<br>**Sex (male, %)** – GNO: (154,80) / PNO: (203,72)	
**Interventions**<br>(Data are presented as median and interquartile ranges)	**Automated pupillometer:**<br>Npi-200 pupillometer® (Neuroptiocs)<br><br>**Targeted temperature management**<br>Target temperature, °C: 33.0 (199, 44%) / 36.0 (256, 56%)<br>Maintenance (hour): 24<br>Rewarming rate (°C/h): not reported<br>TTM devices: not reported<br><br>**Sedation, analgesia, and neuromuscular blocking agents during TTM**<br>Sedation<br>GNO<br>: propofol, 1.7(1.1-2.4) mg / h, midazolam, 0.08(0.04-0.15) mg /kg/ h<br>PNO<br>: propofol, 1.8(1.3-3.7) mg / h, midazolam, 0.11(0.06-0.37) mg /kg/ h<br><br>Analgesia<br>GNO (Fentanyl, 0.49(0.26-1.4) μg/kg/h)<br>PNO (Fentanyl, 0.55(0.14-1.4) μg/kg/h)<br><br>Neuromuscular blocking agents: not reported<br><br>**Supportive care and agent**<br>Norepinephrine (mg/kg/min) - GNO: 4.9 (1–15) / PNO: 5.4 (1–15)<br>Extracorporeal life support (%): not reported	
**Outcomes**	Neurological outcome at 3 months	
**Note**	None	

Quality in Prognosis Studies (QUIPS)		
**Bias**	Author' judgment	Support for judgment
**Study participation**	Low risk	There is adequate participation in the study by eligible individuals
**Study Attrition**	Unclear risk	Reasons for loss to follow-up are not provided.
**Prognostic Factor Measurement**	Low risk	The method and set of measurements of PF are the same for all study participants.
**Outcome Measurement**	Low risk	A clear definition of outcome is provided, including the duration of follow-up and level and extent of the outcome construct.
**Study Confounding**	Unclear risk	Measurement of important confounders is not partially valid and reliable
**Statistical Analysis and Reporting**	Low risk	There is sufficient presentation of data to assess the adequacy of the analysis

Riker 2019	
**Method**	**Type of study: a** single-center prospective observational study<br>**Countries where the study was performed:** USA<br>**Date of trial:** June 28, 2016 - July 22, 2017<br>**Follow-up until:** Neurological outcome: at hospital discharge<br>**Pupillometry measurement time**: hospital day 1
**Participants**<br>(Data are presented as median and interquartile ranges)	**Inclusion:**<br>Encephalopathic adults surviving an initial cardiac arrest (any location or rhythm) were treated with TTM<br><br>**Exclusion:**<br>Not reported<br><br>**The number of participants analyzed:**<br>Good neurological outcome (GNO): 16<br>Poor neurological outcome (PNO): 36<br><br>**Age (years) -** 57 (48-64) in total study population<br>**Sex (male, %)** – 36(65) in total study population
**Interventions**<br>(Data are presented as median and interquartile ranges)	**Automated pupillometer:**<br>Npi-200 pupillometer® (Neuroptiocs)<br><br>**Targeted temperature management**<br>Target temperature, °C: 33.0 in both<br>Maintenance (hour): 24 in both<br>Rewarming rate (°C/h): not reported<br>TTM devices: Arctic Sun (Bard Medical, Covington, GA)<br><br>**Sedation, analgesia, and neuromuscular blocking agents during TTM**<br>Sedation (propofol, 10–30 mcg/kg/min)<br>analgesia (fentanyl, 20–50 mcg/h)<br>neuromuscular blocking agents: not reported<br><br>**Supportive care and agent**<br>Norepinephrine (mg/day): not reported<br>Extracorporeal life support (%): not reported

**Outcomes**	Neurological outcome at hospital discharge	
**Note**	None	
**Quality in Prognosis Studies (QUIPS)**		
**Bias**	Author' judgment	Support for judgment
**Study participation**	Unclear risk	The baseline study sample is not adequately described for key characteristics (LIST).
**Study Attrition**	Low risk	Participants lost to follow-up are adequately described for key characteristics (LIST).
**Prognostic Factor Measurement**	Unclear risk	The method and setting of measurement are not the same for all study participants.
**Outcome Measurement**	Low risk	A clear definition of outcome is provided, including the duration of follow-up and level and extent of the outcome construct.
**Study Confounding**	High risk	Important potential confounders are not accounted for in the study design
**Statistical Analysis and Reporting**	Low risk	There is sufficient presentation of data to assess the adequacy of the analysis.

Abbreviations: TTM = target temperature management GNO; PF = prognostic factor.

Table S7. Data abstraction and study quality assessment of included studies

Study	Outcome, Time point	Cut-off (%)	TP (n)	FP (n)	FN (n)	TN (n)	Sensitivity (%)	Specificity (%)	PPV (%)	NPV (%)	AUC
Suys 2014	3 months	13	18	2	9	21	66.7	91.3	90	70	0.79
Heimburger 2016	3 months	9	35	6	20	21	63.6	77.8	85.3	51.2	0.76
Riker 2019	Hospital discharge	5	16	1	20	15	44.4	93.7	94.1	42.8	0.75

Abbreviations: TP = true positive; FP = false positive; FN = false negative; TN = true negative; PPV = positive predictive value; NPV = negative predictive value; AUC = area under the curve.

Table S8. Data abstraction and study quality assessment of included studies

Author(s):
Question: In the post-cardiac arrest patients treated with TTM, patients with higher %PLR will have better neurological outcomes compared to patients with lower %PLR ?
Setting:
Bibliography:

Certainty assessment							№ of patients		Effect		Certainty	Importance
№ of studies	Study design	Risk of bias	Inconsistency	Indirectness	Imprecision	Other considerations	patients with higher %PLR	patients with lower %PLR	Relative (95% CI)	Absolute (95% CI)		
**Good neurological outcome at 3 months (%PLR measured at 0-24 h)**												
3	observational studies	not serious	not serious	not serious	not serious [a, b]	none	231	335	-	SMD **0.87 SD higher** (0.7 higher to 1.02 higher)	⨁⨁◯◯ Low	CRITICAL
**Good neurological outcome at 3 months (%PLR measured at 24-48 h)**												
3	observational studies	not serious	not serious	not serious	not serious	none	187	290	-	SMD **0.86 SD higher** (0.4 higher to 1.32 higher)	⨁⨁◯◯ Low	CRITICAL

CI: confidence interval; **SMD:** standardized mean difference

Explanations
a. %PLR: percent constriction of the pupil light reflex
b. TTM: targeted temperature management

Table S9. Quantitative pupillary parameters of automated pupilometer in the included studies

Authors	Assessment time after admission	Pupillometer	Pupillary Parameter	The time point of neurological outcome
Suys	0-24h and 24-48h	NeuroLight Algiscan® (IDMED, Marseille, France)	Quantitative PLR	At 3 months
Heimburger	0-24h and 24-48h	NeuroLight Algiscan® (IDMED, Marseille, France)	Quantitative PLR<br>Latency duration<br>Constriction velocity	At 3 months
Oddo	0-24h, 24-48h and 48-72h	Npi-200 pupillometer® (Neuroptics)	Quantitative PLR<br>NPi	At 3 months
Riker	0-24h	Npi-200 pupillometer® (Neuroptics)	Quantitative PLR<br>NPi	At hospital discharge

Abbreviation: NPi = neurological pupil index; PLR=pupillary light reflex

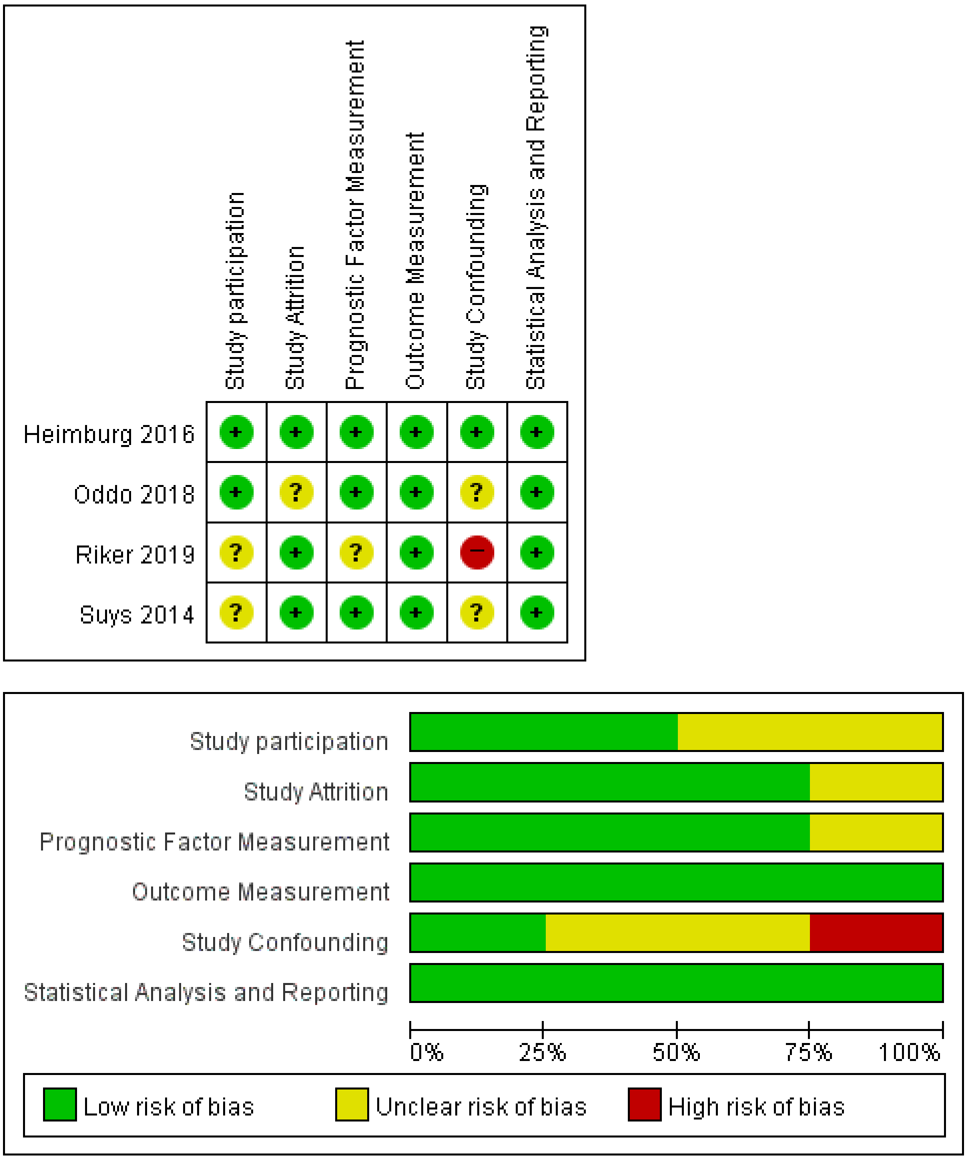

Figure. S1 Summary of assessment of the risk of bias in the included studies

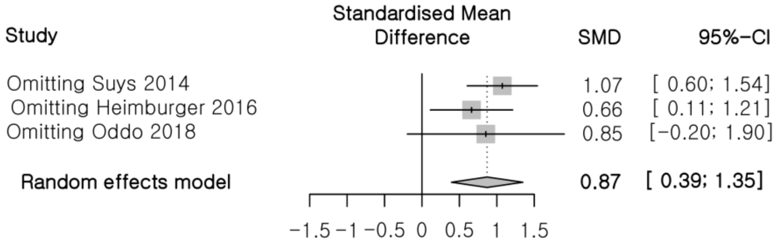

Figure. S2 the sensitivity analysis of the included studies
Abbreviations: CI, confidence interval; SD, standard deviation

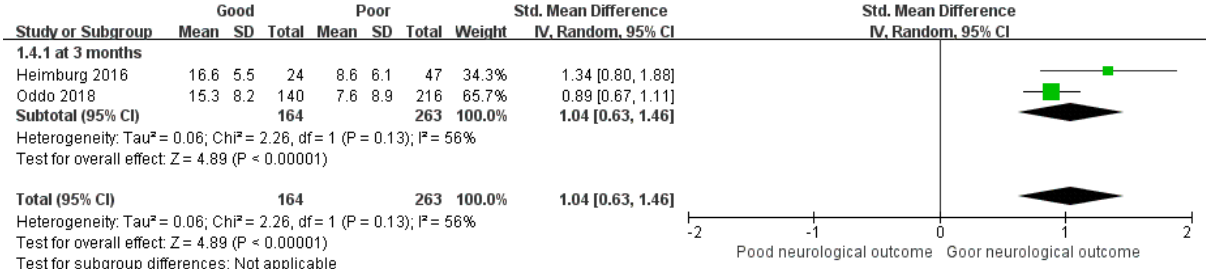

Figure. S3 Forest plot for percent constriction of pupillary light reflex measured at 24–48 h after hospital admission and neurological outcome at 3 months after the sensitivity analysis of the included studies
Abbreviations: CI, confidence interval; SD, standard deviation

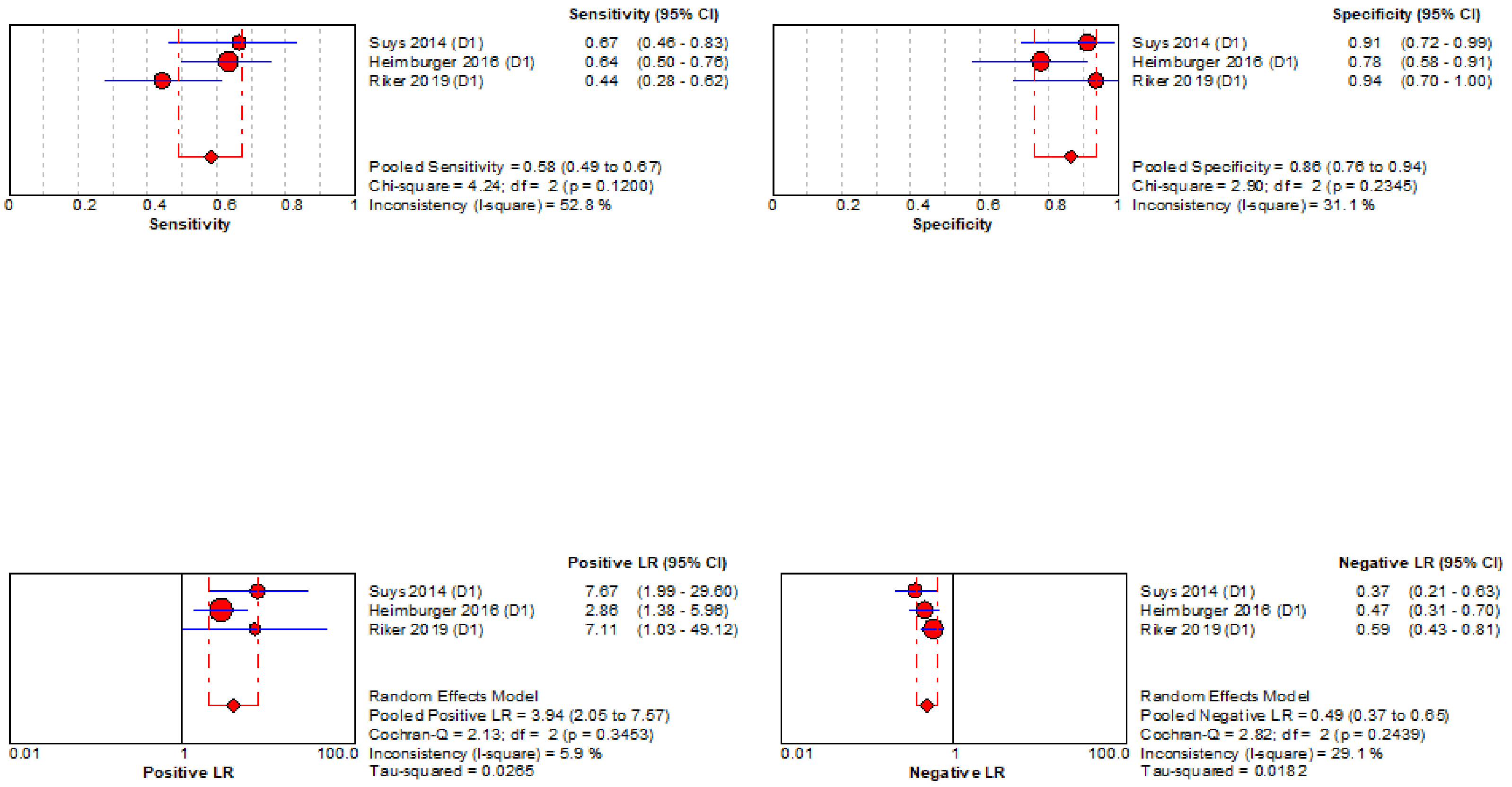

Figure. S4 Pooled prognostic accuracy of the percent constriction of pupillary light reflex for poor neurological outcome.
A: Pooled sensitivity, B: pooled specificity, C: pooled positive likelihood ratio, D: pooled negative likelihood ratio
Abbreviations: CI, confidence interval; LR, likelihood.
